# Spatio-Temporal Characterization of Brain Inflammation in a Non-human Primate Stroke Model Mimicking Endovascular Thrombectomy

**DOI:** 10.1007/s13311-023-01368-2

**Published:** 2023-03-28

**Authors:** Guillaume Becker, Justine Debatisse, Margaux Rivière, Claire Crola Da Silva, Maude Beaudoin-Gobert, Omer Eker, Océane Wateau, Tae Hee Cho, Marlène Wiart, Léon Tremblay, Nicolas Costes, Inès Mérida, Jérôme Redouté, Christelle Léon, Jean-Baptiste Langlois, Didier Le Bars, Sophie Lancelot, Norbert Nighoghossian, Laura Mechtouff, Emmanuelle Canet-Soulas

**Affiliations:** 1 CarMeN Laboratory, INRAE U1397, INSERM U1060, Groupement Hospitalier Est, University Claude Bernard Lyon 1, 59 Boulevard Pinel, 69500 Lyon, Bron, France; 2 Lyon Neuroscience Research Center, UMR5295, INSERM U1028, CNRS, Université Claude Bernard Lyon 1, Lyon, France; 3 UMR-5220, CREATIS, CNRS, INSERM U1206, Université Lyon 1, INSA Lyon, Villeurbanne, France; 4 Hospices Civils de Lyon, Lyon, France; 5 Cynbiose SAS, Lyon, France; 6 Cognitive Neuroscience Center, CNRS UMR5229, Université Claude Bernard Lyon 1, Lyon, France; 7 CERMEP, Lyon, France

**Keywords:** Stroke, Inflammation, Endovascular thrombectomy, PET-MRI, TSPO

## Abstract

Reperfusion therapies in acute ischemic stroke have demonstrated their efficacy in promoting clinical recovery. However, ischemia/reperfusion injury and related inflammation remain a major challenge in patient clinical management. We evaluated the spatio-temporal evolution of inflammation using sequential clinical [^11^C]PK11195 PET-MRI in a non-human primate (NHP) stroke model mimicking endovascular thrombectomy (EVT) with a neuroprotective cyclosporine A (CsA) treatment. The NHP underwent a 110-min transient endovascular middle cerebral artery occlusion. We acquired [^11^C]PK11195 dynamic PET-MR imaging at baseline, 7 and 30 days after intervention. Individual voxel-wise analysis was performed thanks to a baseline scan database. We quantified [^11^C]PK11195 in anatomical regions and in lesioned areas defined on per-occlusion MR diffusion-weighted imaging and perfusion [^15^O_2_]H_2_OPET imaging. [^11^C]PK11195 parametric maps showed a clear uptake overlapping the lesion core at D7, which further increased at D30. Voxel-wise analysis identified individuals with significant inflammation at D30, with voxels located within the most severe diffusion reduction area during occlusion, mainly in the putamen. The quantitative analysis revealed that thalamic inflammation lasted until D30 and was significantly reduced in the CsA-treated group compared to the placebo. In conclusion, we showed that chronic inflammation matched ADC decrease at occlusion time, a region exposed to an initial burst of damage-associated molecular patterns, in an NHP stroke model mimicking EVT. We described secondary thalamic inflammation and the protective effect of CsA in this region. We propose that major ADC drop in the putamen during occlusion may identify individuals who could benefit from early personalized treatment targeting inflammation.

**Supplementary Information:**

The online version contains supplementary material available at 10.1007/s13311-023-01368-2.

## Introduction

Intravenous thrombolysis and, in cases of large vessel occlusion, endovascular thrombectomy (EVT) are the current standard-of-care of acute ischemic stroke (AIS) to promote clinical recovery [1]. However, more than half of patients treated by EVT remain disabled despite successful reperfusion [2, 3]. The pathological mechanisms underlying these cases of “futile reperfusion” are incompletely understood, but cerebral ischemic reperfusion (CIR) damage and the related inflammation may contribute to these mechanisms [4–6]. This phenomenon has already been widely studied in rodent models, but the dynamic of inflammation remains far from being fully understood in the gyrencephalic brain [7]. Inflammation is mediated by a variety of factors and genes, and cellular substrates such as microglia are still questioned regarding their detrimental or beneficial aspects [8, 9] and their loco-regional modulation in gray and white matter tissue is also not well described [10]. The eligibility criteria and the therapeutic window for immunomodulatory treatments remain critical issues to set up CIR clinical trial, which have so far failed to improve outcome [11], and robust companion imaging biomarkers are therefore essential [12].

A better knowledge of this process and assessment of novel neuroprotective therapies targeting inflammation in combination with reperfusion strategies will highly benefit from experimental translational models that allow therapeutic evaluations. In this context, we previously developed a model of transient occlusion of M2 segment of the middle cerebral artery (MCA) mimicking EVT in *Macaca fascicularis*. We performed per-occlusion and per-recanalization simultaneous PET-MR imaging to assess the infarct core and the cerebral penumbra, and to define quantitative imaging metrics for further therapeutic evaluation as well [13].

Furthermore, we investigated blood–brain barrier (BBB) dysfunction, measured by dynamic contrast-enhanced (DCE) MRI, which is currently considered one of the major contributors to CIR injuries [11]. We used PET-MRI and hybrid nanoparticles [^68^ Ga]AGuIX to assess the protective effect of a single injection of cyclosporine A on BBB permeability in our NHP model of stroke. Cyclosporine A has been shown to prevent BBB breakdown by blocking the cyclophilin A pathway in the pericytes and thereby protecting the neuro-vascular unit [14, 15]. Besides the reduction of hyperpermeability induced by CsA at the hyperacute phase, the permeability was also preserved in the choroid plexuses and the early burst of circulating MMP-9 was limited compared to non-treated animals [16].

In the present study, we characterize post-EVT inflammation in our NHP model of CIR with a clinical imaging protocol (designed with per-occlusion [^15^O]H_2_O PET-MR imaging to assess the core and the ischemic penumbra). We performed longitudinal PET-MR imaging with the 18 kDa translocator protein (TSPO) PET radiotracer [^11^C]-(*R*)-PK11195 (index of microglial reactivity). Our primary objective was to characterize spatio-temporal evolution of CIR-induced inflammation in our NHP model. We computed a [^11^C]-(*R*)-PK11195 baseline database for individual voxel-wise analysis follow-up to identify subjects with overreaching inflammation. Moreover, we performed analysis of the microglial response both in the lesion core and the ischemic penumbra as well as throughout the entire brain thanks to an atlas-based approach. The secondary objective was to evaluate the impact on brain inflammation of an acute CsA treatment used as pharmacological protector of the neuro-vascular unit at the time of reperfusion.

## Materials and Methods

### Animals

All experiments were carried out in accordance with the European Directive 2010/63/UE, approved by the Ethics Committee and authorized by the Ministry of Higher Education, Research and Innovation (APAFIS#4702 and 8901).

The study included mature male cynomolgus macaques (*Macaca fascicularis*) sourced from Mauritius. The study complied with the ARRIVE guidelines [17], including critical examination for inclusion/exclusion criteria, randomization for treatment group allocation, and blinded analysis of treatment at all time points. We include in the database all the 18 animals we scanned at baseline with (*R*)-[^11^C]PK11195. These baseline scans were acquired through several studies, 4 of them were published in di Cataldo et al. [18]. and the 14 others were presented in our previous study on the EVT model in *M. fascicularis* [13, 16]. These 14 cases are reported in the present study as part of the cohort of 16 animals which were included in the study if they underwent successful MCA occlusion (MCAo), assessed visually using per-occlusion X-ray angiography.

### Experimental Design

Experimental design of all imaging examination, including per-occlusion and post-recanalization, was previously described [13]. The schematic representation is reproduced in Supplemental Fig. 1. Five minutes before recanalization of the occluded MCA, cyclosporine A (CsA, 2 mg/kg, Sandimmum, Novartis, 50 mg mL^−1^, diluted in saline to 3.33 mg mL^−1^) or NaCl were injected via an intravenous catheter.

**Fig. 1:**
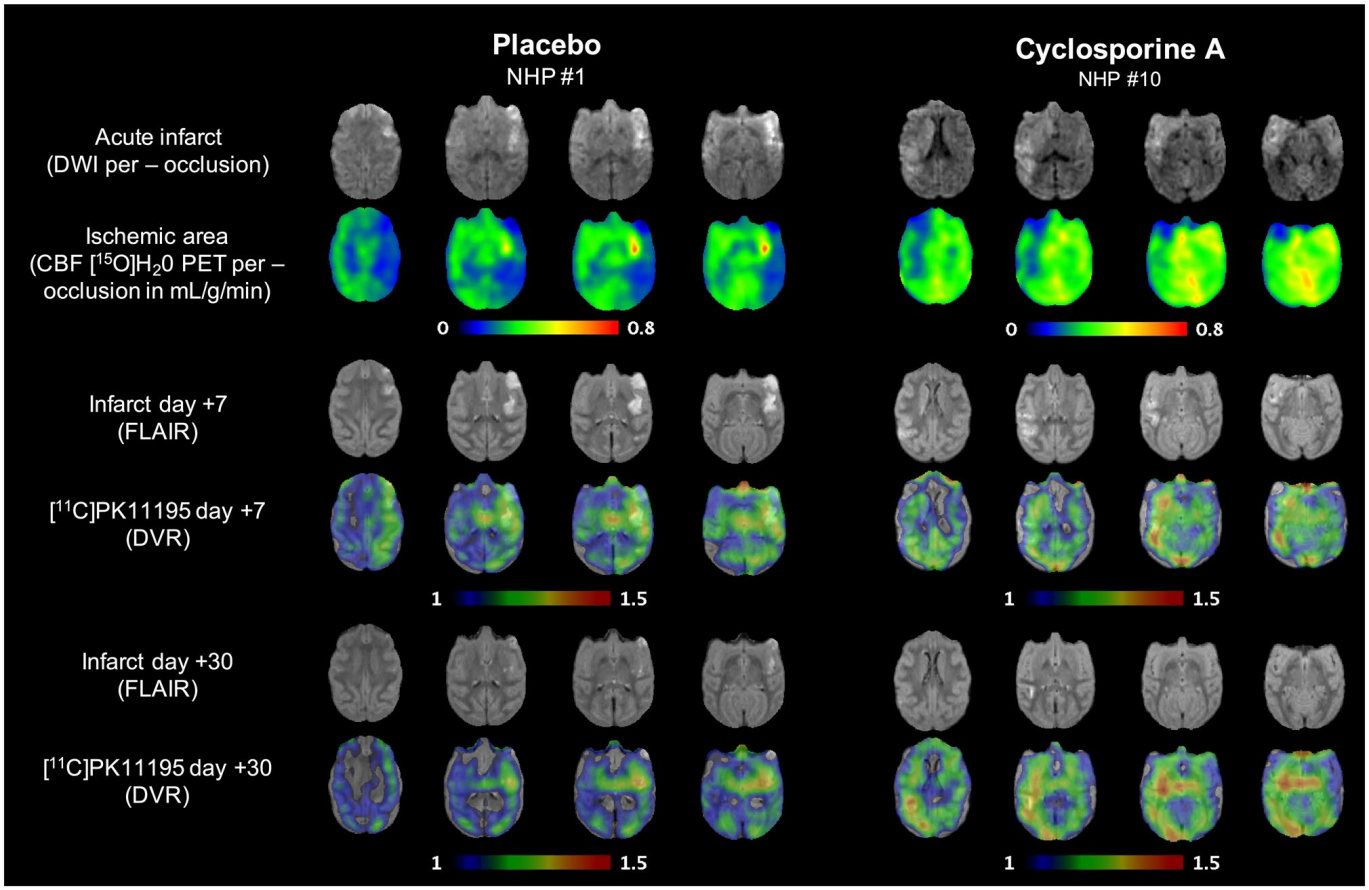
Example of individual dataset from each group of the study. From top to bottom: diffusion-weighted imaging per-occlusion and [^15^O]H_2_O perfusion PET per-occlusion; fluid attenuation inversion recovery MRI at day 7 and overlay of [^11^C]PK11195 DVR; fluid attenuation inversion recovery MRI at day 30 and overlay of [^11^C]PK11195 DVR

### Animal Model and Endovascular Procedure

Experimental anesthesia and endovascular procedure were presented in details previously [13, 16]. Briefly, middle cerebral artery occlusion (MCAo) was induced in 7-year-old mature male *Macaca fascicularis* for 110 min by an endovascular coil under sevoflurane anesthesia and continuous monitoring. PET-MR imaging was performed during occlusion, after coil removal (post-recanalization) and at day 7 and day 30 after ischemia reperfusion.

### [^11^C]PK11195 Radiochemistry

(*R*)-[^11^C]PK11195 (thereafter referred as [^11^C]PK11195) was prepared by N-methylation of the desmethyl precursor in DMSO at room temperature, via [^11^C]methyl-iodide and HPLC [19, 20], either with iPhase C-11 Pro or with Scansys synthesis modules. Following the synthesis of [^11^C]PK11195, the radioligand was obtained after SPE in 10% ethanol sterile saline solution and filtered through a sterile Millex-GV filter (0.22 μm; Millipore).

### Imaging Data Acquisition and Processing

All imaging data were acquired on the fully integrated hybrid Biograph mMR PET-MRI scanner (Siemens Healthcare, Erlangen, Germany) at baseline (several weeks before the vascular procedure), per-occlusion and post-revascularization, and at days 7 (D7) and 30 (D30) after revascularization (Supplemental Fig. 1).

### PET-MRI Evaluation of Stroke Lesions

Imaging data protocol was presented previously [13, 16]. Briefly, it consists of state-of-the-art clinical stroke MRI with time-of-flight (TOF) angiography, diffusion-weighted imaging (DWI), perfusion weighted imaging (dynamic susceptibility contrast, DSC with Gadolinium-DOTA bolus injection), T2*, and T2 FLAIR. It was combined with PET imaging where dynamic brain perfusion PET data were acquired over 6 min after bolus injection of radiotracer [^15^O]H_2_O (255 ± 15 MBq) followed by i.v. injection of 10 mL saline (injection rate, 3 mL/s) using the power injector. Using DWI at occlusion, the acute core lesion was segmented by an experienced neurologist with a semi-automated method (3D Slicer, https://www.slicer.org) by using both a validated apparent diffusion coefficient (ADC) threshold (ADC < 620 × 10^−6^ mm^2^/s) and visual assessment of b1000 images. The ischemic zone was defined using [^15^O]H_2_O PET and the established threshold < 20 mL/min/100 g. BBB disruption was assessed in each subject using DCE maps as well as post-gadolinium FLAIR. T2 FLAIR at day 7 was used to measure the infarct volume. MRI analysis was done by a senior neurologist to segment acute core lesion and FLAIR established infarct lesion. We previously described the significant contribution of post-recanalization progression volume which account for 45% of the final infarct size. This lesion’s progression was mainly located in the temporal cortex and related temporal limbic structures [13]. Besides, the BBB permeability, quantified through the transfer constant *K*_trans_ of AGuIX® nanoparticles and PET imaging, was enhanced after EVT in the ischemic area at early reperfusion time [16].

### PET Evaluation of Inflammation

Additionally to [^15^O]H_2_O PET acquisitions, the PET-MRI sessions included a dynamic brain PET scan for the baseline session, and at 7 and 30 days post-EVT. Data were acquired over 70 min in list-mode format after bolus injection of radiotracer [^11^C]PK11195 (mean injected activity 139 ± 20 MBq, range 82–183 MBq) followed by i.v. injection of 10 mL saline (injection rate, 3 mL/s) using the power injector. The data were reconstructed on a 256 × 256 × 127 matrix (voxel size: 0.7 × 0.7 × 2.03 mm^3^), 26 cm FoV using a point-spread function and OP-OSEM iterative reconstruction method including normalization as well as correction for attenuation, scatter, random counts, and dead time. Prior to the PET-MRI session, a CT scan (Siemens Biograph mCT64, Siemens Healthcare, Erlangen, Germany) was obtained for each animal and used for PET attenuation correction. Dynamic PET data were reconstructed in 28 frames: 6 × 10 s, 6 × 20 s, 6 × 120 s, and 8 × 300 s. [^11^C]PK11195 PET data analysis was done using supervised clustering analysis for reference region determination (5th version, V University Medical Center (VUMC) Amsterdam, The Netherlands). The distribution volume ratios (DVR) were computed as outcome parameters thanks to Logan kinetic modeling [21, 22]. The final outcomes were the parametric DVR maps.

### Database Post-Processing and Validation

Post-processing pipeline is summarized in Supplemental Fig. 2. Baseline [^11^C]PK11195 DVR maps were spatially normalized in a common *M. fascicularis* template space [13, 23] then smoothed with a Gaussian filter of 2 × 2 × 2 mm. The database of baseline scans was validated using a “leave-one-out” method where each individual scan was tested against the others on a voxel-wise analysis with SPM12 using the 2-sample *t*-test design (ANCOVA with global mean normalization).

**Fig. 2:**
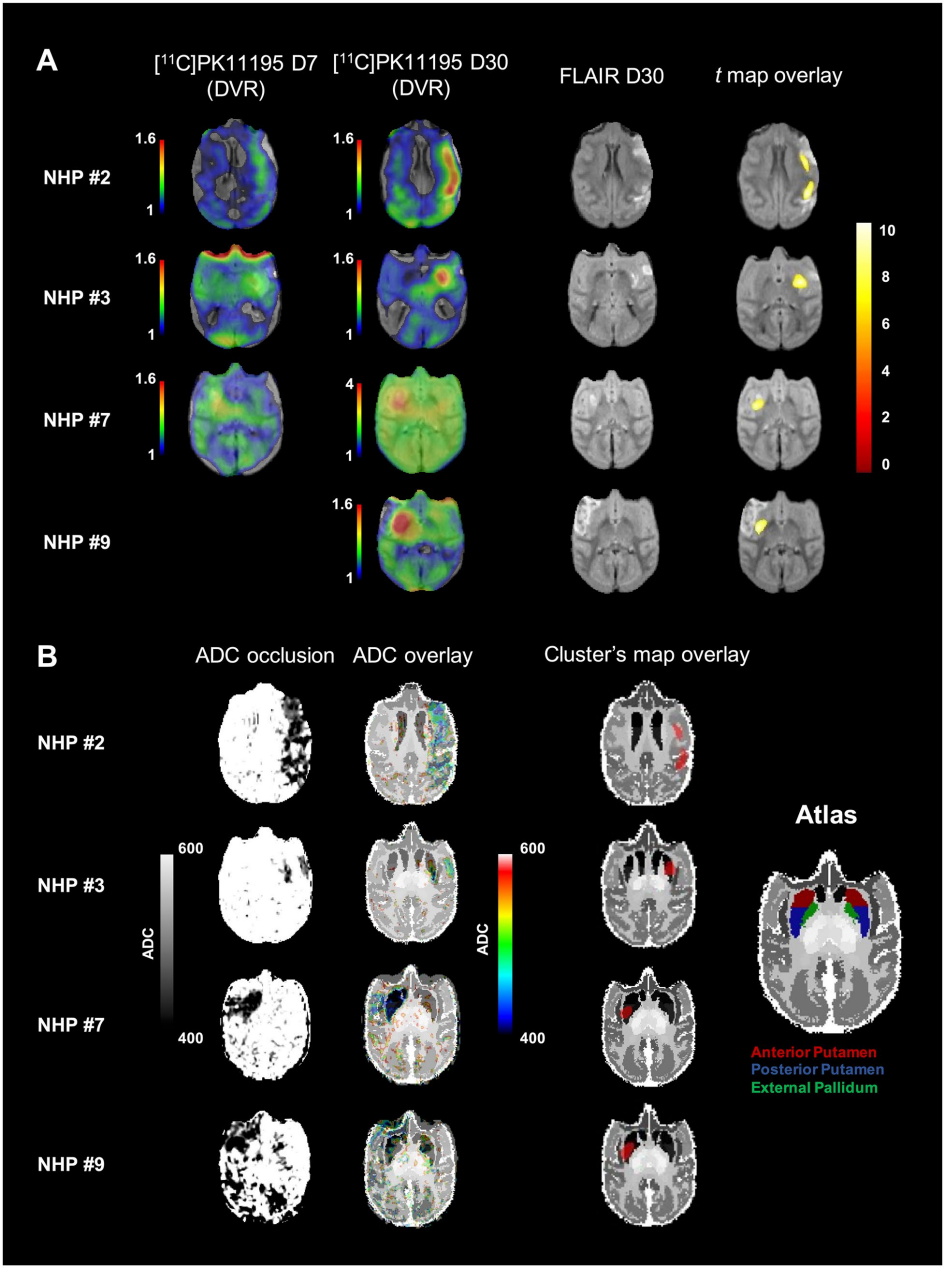
**A** SPMs showing the spatial distribution of significant voxels (*p* < 0.05 corrected) where [^11^C]PK11195 DVR increase after MCAO in NHP compared to the baseline database. The first column represents the corresponding [^11^C]PK11195 DVR map at day 30 (see color scale on the left), the second one the individual FLAIR post-gadolinium imaging at day 30. Clusters of significant voxels are superimposed on corresponding individual FLAIR post-gadolinium imaging at day 30 (third column). *Z* score in color scale on the right. **B** ADC maps during occlusion of the four individuals identified by persistent inflammation. The first column represents the ADC maps at occlusion (with the corresponding color scale). ADC maps are superimposed on *M. fascicularis* atlas in the second column. The third column displayed the localization of SPM identified clusters of [^11^C]PK11195 DVR increase at D30 compared to baseline database

The day 7 and day 30 [^11^C]PK11195 DVR maps were processed similarly and then tested individually against the baseline database on a voxel-wise analysis with SPM12. For all statistical inferences, we set a significant cluster threshold at 100 voxels and statistical threshold at *p* < 0.05 FWE corrected at the cluster level.

### Region-Based Measurement

DVRs were extracted from the lesion core and the penumbra as well as from *M. fascicularis* brain atlas regions [23]. Results were expressed as actual differences in DVR between the baseline and day 7 (Δ DVR_D7−baseline_) and between the baseline and day 30 (Δ DVR_D30−baseline_).

### Immunohistochemistry

#### Tissue Processing

After the last PET-MR session, animals were deeply anaesthetized with ketamine (10 mg/kg, i.m.) and sacrificed by lethal injection at the end of the experiment. After carotid perfusion (2 L NaCl, 0.9‰), the brains were removed and stored in 4% paraformaldehyde at 4 °C until sectioning which was performed on a cryostat microtome (CM3050 S, LEICA Biosystem). After 3 washes of 5 min in phosphate buffer saline (PBS), the brain samples were immersed in cooled isopentane and cut at 30 μm of thickness. Brain slices were freshly mounted on slides and store at −80 °C until use.

On the day of experiment, the slides were allowed to warm up at room temperature. Then, the brain slices were permeabilized 60 min in PBS containing 0.1% Tween 20 (Sigma-Aldrich) and 0.3% Triton X-100 (Sigma-Aldrich) and 5% of bovine serum albumin (BSA); afterward, blocking was performed for 30 min in PBS containing 0.1% Tween 20 and 5% of BSA (PBS-Tween-BSA). The sections were then incubated overnight at 4 °C with the primary antibodies diluted in the same PBS-Tween-BSA (Supplemental Table 1). After 3 washes of 5 min in PBS-Tween, they were incubated for 2 h at room temperature with secondary antibody diluted in PBS-Tween-BSA, rinsed 3 times for 5 min in PBS-Tween, and finally mounted on glass slides using an antifade mounting medium with 4′,6-diamidino-2-phenylindole (Vectashield® Hardset™, Vector Laboratories).

**Table 1:** Summary of demographical and lesion data per animal

	Weight (kg) *				**Occlusion**				**Reperfusion**		**Lesion volumes**		**Voxel-wise analysis**		
**NHP ID**	**Baseline**	**Day 7**	**Day 30**	**Treatment**	**Occ. side**	**DWI lesion core (mL)**	**CBF (mL)**	**PET penumbra (mL)**	**DWI lesion core (mL)**	**CBF (mL)**	**FLAIR D7 (mL)**	**FLAIR D30 (mL)**	**Database**	**Day 7**	**Day 30**
NHP #1	7.3	8.4	8.2	Placebo	Left	2.7	13.4	11.9	2.2	7.5	2.9	0.3	incl	incl	incl
NHP #4	9.7	11.6	10.9	Placebo	Left	4.2	2.4	1.0	1.0	0.0	2.9	1.4	incl	incl	incl
NHP #6	7.6	8.3	8.5	Placebo	Left	0.6	2.3	2.0	0.0	0.0	0.2	0.0	incl	incl	incl
NHP #7	6.8	6.5	6.6	Placebo	Right	3.4	18.0	15.0	1.6	5.6	0.5	0.1	incl	incl	incl
NHP #9	7.3	-	7.5	Placebo	Right	6.6	5.1	1.7	3.6	0.4	6.4	3.3	incl	-	incl
NHP #12	-	5.3	6.1	Placebo	Right	7.7	2.7	0.9	9.0	0.1	4.6	3.9	-	incl	incl
NHP #13	7.1	7.2	7.5	Placebo	Right	2.3	1.2	0.9	2.3	0.0	1.8	0.5	incl	incl	incl
NHP #16	7.1	-	-	Placebo	Right	3.5	0.2	0.2	7.5	3.2	-	-	incl	-	-
Mean	7.5	7.9	7.9			3.9	5.7	4.2	3.4	2.1	2.8	1.4			
SD	1.0	2.2	1.6			2.3	6.5	5.8	3.2	3.0	2.2	1.6			
NHP #2	10.1	10.4	9.5	CsA	Left	8.2	8.1	3.1	4.7	6.9	4.4	2.7	incl	incl	incl
NHP #3	8.6	8.5	8.8	CsA	Left	3.9	0.8	0.4	2.7	1.3	3.1	0.4	incl	incl	incl
NHP #8	6.9	6.6	6.8	CsA	Left	2.2	1.9	1.1	1.8	0.2	0.6	0.0	incl	incl	incl
NHP #10	6.8	6.9	7.2	CsA	Right	4.4	3.4	2.4	4.4	0.0	0.7	0.1	incl	incl	incl
NHP #11	7.7	7.6	7.7	CsA	Left	0.5	0.4	0.4	0.4	0.8	0.1	0.0	incl	incl	incl
NHP #14	6.7	-	-	CsA	Left	11.6	-	-	8.2	-	-	-	incl	-	-
NHP #15	-	6.5	7.1	CsA	Right	0.1	0.0	-	2.1	0.0	0.0	0.0	-	incl	incl
Mean	7.8	7.7	7.8			4.4	2.1	1.1	3.5	1.5	1.5	0.5			
SD	1.3	1.5	1.1			4.2	2.9	1.2	2.5	2.7	1.8	1.1			

Occlusion, reperfusion, and lesion volumes were previously published in Debatisse et al. (*JCBFM* 2021) [13] and Debatisse et al. *Brain Comm* (2020) [16]

*Occ. side* occlusion side, *Incl.*: included in voxel-wise analysis

^*^The weight was measured at the time of each [^11^C]PK11195 PET scans (the absence of weight indicates an absence of [^11^C]PK11195 PET scan)

#### Histological Image Analysis

Images were obtained using a Zeiss Axioscan Z1 slice scanner equipped with 20 × objective and the Zeiss software (Carl Zeiss AG, Germany). Images were initially processed using Module Macro Environment in Zen Blue 2.3 (Zeiss) to correct scanner aberrations.

### Statistical Analysis

All values are presented as median and interquartile range [first quartile: Q1, third quartile: Q3]. Non-parametric Mann–Whitney test was used to compare the Δ DVRs between the placebo and the CsA-treated groups. Statistical significance threshold was set at 0.05. All statistical analyses were performed using the Prism GraphPad software, version 6 (GraphPad Software, La Jolla, CQ, USA).

## Results

Sixteen animals with a follow-up with inflammation imaging at day 7 and day 30 were included in the study’s cohort. Two animals died during or shortly after the EVT: 1 from the CsA group was a fast progressor with hemorrhage (NHP #14) and 1 from the placebo for which the recanalization failed due to a defective coil (NHP #16). Two animals were excluded due to an absence of [^11^C]PK11195 baseline scan (NHP #12 and #15), and one animal (NHP #09) was excluded because of fail in [^11^C]PK11195 D7 scan. Final inclusions in placebo and CsA groups were 6 and 5 animals respectively. Table 1 summarizes animals’ data for the database and voxel-wise analyses, alongside with the individual lesion’s data, measured using DWI-MRI and [^15^O]H_2_O PET during the occlusion reperfusion, and FLAIR MRI during the occlusion reperfusion and the follow-up respectively.

### Visual Assessment of [^11^C]PK11195 PET Evolution from D7 to D30

Figure 1 illustrates representative maps of [^11^C]PK11195 for both the CsA and placebo group. In both groups, [^11^C]PK11195 DVR maps highlighted a focal increase in [^11^C]PK11195 uptake overlapping the lesion core, assessed by FLAIR MRI at day 7. In addition to this localized increase, we observed a diffuse [^11^C]PK11195 uptake that occupied nearly the entire brain. The inflammatory process also involved brain regions contralateral to the lesion side with DVR increase in both the contralateral hemisphere (cortical and subcortical regions) and the cerebellum. This global diffuse uptake was independent from the infarct size. This inflammatory pattern showed a declining trend over time but remains present in 81% of the animals (9 over 11). At day 30, the majority of the animals present a pattern of sustained focal inflammation, sometime alongside with a lower diffuse [^11^C]PK11195 uptake (Supplemental Fig. 3 for additional DVR maps).

**Fig. 3:**
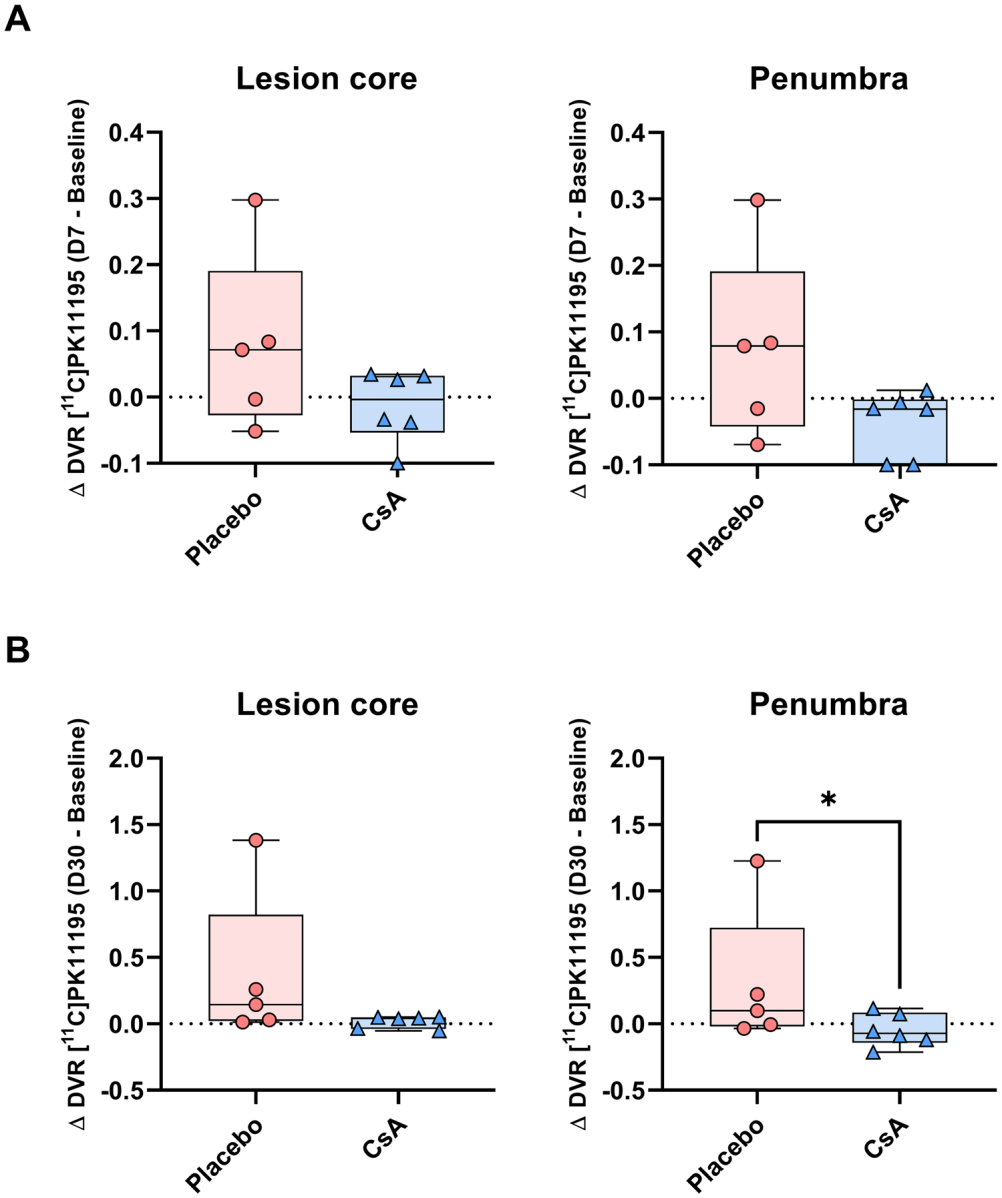
Quantification in lesion core and penumbra. Individual values of absolute differences in [.^11^C]PK11195 DVR. **A** Differences between the baseline and day 7 (Δ DVR_D7−baseline_). **B** Differences between the baseline and day 30 (Δ DVR_D30−baseline_). Placebo *n* = 5, CsA *n* = 6. Non-parametric Mann–Whitney test, * *p* < .05

### [^11^C]PK11195 Voxel-Wise Analysis for Clusters’ Detection

The “leave-one-out” validation method has not identified any anomalies between the NHP [^11^C]PK11195 baseline scans. Individual DVR at day 7 and day 30 were compared to the baseline DVR database composed of 17 volumes. At the day 7 timepoint, SPM12 did not show voxel clusters of significant differences. However, at the day 30 timepoint, 4 animals (36%, NHP #2, #3 and NHP #7, #9 in the CsA and placebo groups respectively) displayed significantly increased DVR clusters compared to baseline database. These voxel clusters were located in the region of focal inflammation of the corresponding [^11^C]PK11195 DVR maps (Fig. 2, panel A). The corresponding voxels, *z* scores, and peak coordinates are reported in Table 2. There were no significant clusters detected in the rest of the brain. To identify the regions involved, we mapped the cluster’s mask of the four animals over the *M. fascicularis* atlas to identify the regions involved (Fig. 2, panel B). In 3 out of 4 individuals (NHP #3, #7, and #9), the cluster led mainly in the posterior putamen, whereas only the NHP #2 displayed clusters in cortical areas. Finally, we compared these cluster’s masks to the lesion’s and the penumbra’s masks (Supplemental Fig. 4). We found that the cluster’s masks were located in the lesion core defined by DWI-MRI during the occlusion. Figure 2 represented ADC maps overlay on *M. fascicularis* atlas and showed the lowest ADC values in the posterior putamen for NHP #3, #7, and #9, corresponding to the cluster localization.

**Table 2:** Voxel-wise results at day 30

		**Cluster**				**Coordinates**		
**Timepoint**	**NHP ID**	*p*(FWE-corr)	Equiv. K (mm^3^)	*p*(unc)	*Z* score	*x*	*y*	*z*
Day 30	NHP #2	0	1440 (317)	0	5.76	−19	−20.9	8.5
		0	930 (205)	0	5.56	−17.8	−2.2	7.9
	NHP #3	0	1328 (292)	0	5.82	−13	−2.8	2.5
	NHP #7	0	1034 (228)	0	5.55	14	−4.6	0.7
	NHP #9	0	1214 (266)	0	5.46	14.6	−5.8	2.5

Statistical results of the voxel-wise analysis. *p*(FWE-corr): *p* value (family-wise error corrected, < .05). equiv. k: number of voxels in the cluster corresponding volume in mm^3^. *p*(unc): *p* value uncorrected (< .001). *Z* score of the significant cluster. Coordinates: peak’s coordinates in the template space

**Fig. 4:**
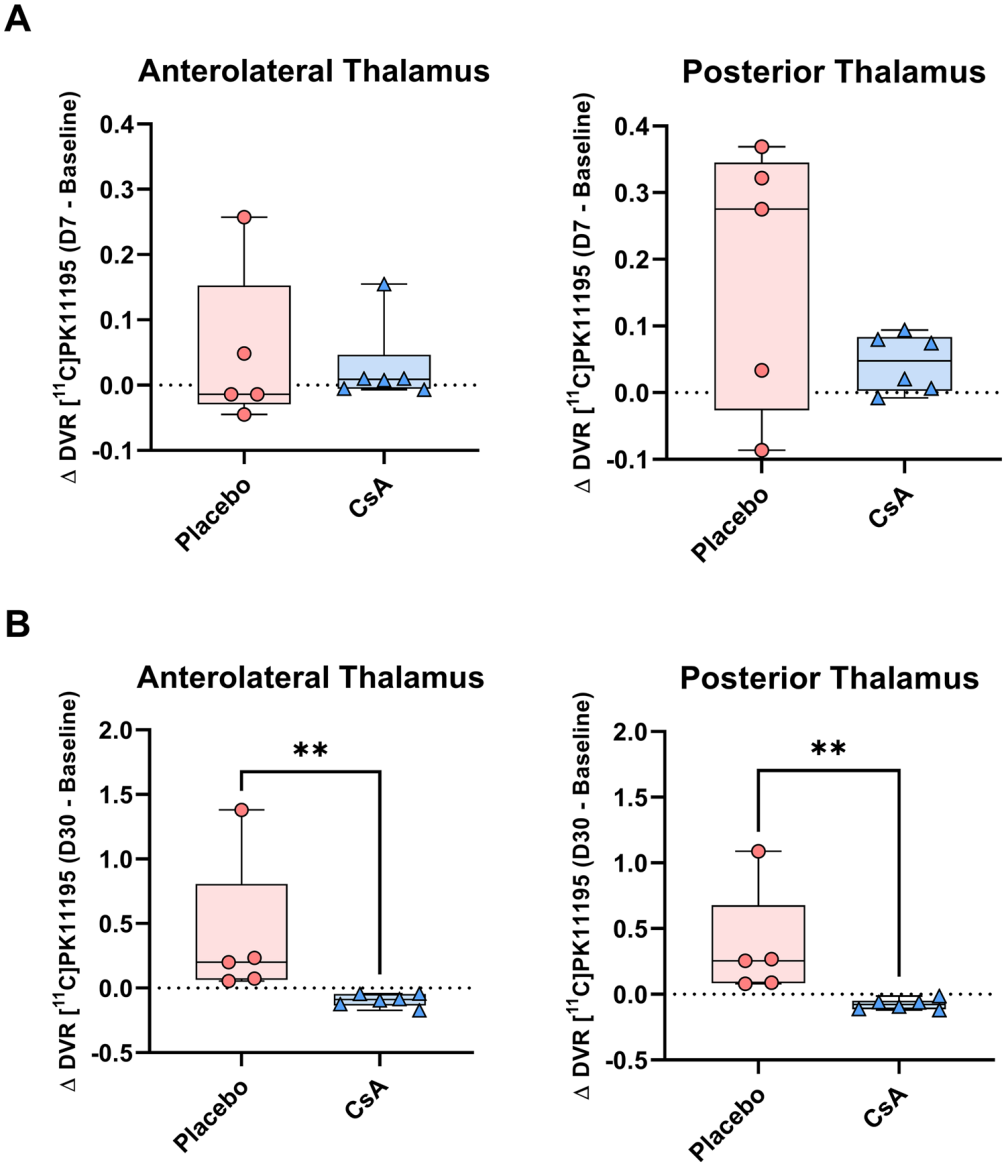
Quantification in the thalamus. Individual values of absolute differences in [^11^C]PK11195 DVR. **A** Differences between the baseline and day 7 (Δ DVR_D7−baseline_). **B** Differences between the baseline and day 30 (Δ DVR_D30−baseline_). Placebo *n* = 5, CsA *n* = 6. Non-parametric Mann–Whitney test, ** *p* < .05

### [^11^C]PK11195 PET Quantification of Spatio-Temporal Changes Compared to Baseline

The differences (Δ DVR_D7−baseline_) and (Δ DVR_D30−baseline_) were computed in the lesion core and penumbra. Figure 3 shows a tendency to higher Δ DVR_D7−baseline_ values and variability in the placebo group compared to the CsA-treated group not only in the lesion core but also in the penumbra (lesion core: 0.071 [−0.027, 0.190] vs. −0.004 [−0.053, 0.032] for placebo group vs. CsA-treated group respectively; penumbra: 0.079 [−0.042, 0.190] vs. −0.016 [−0.017, −0.001] for placebo group vs. CsA-treated group respectively). We observe that the lesion core and the penumbra also showed the same pattern for the Δ DVR_D30−baseline_ with similar values. Non-parametric Mann–Whitney test revealed significant differences in the penumbra at day 30 between the placebo group and the CsA-treated group (0.099 [0.019, 0.722] vs. −0.073 [−0.140, −0.084] respectively, *p* = 0.041).

The [^11^C]PK11195 was analyzed in brain anatomical regions thanks to atlas-based ROIs. In most of them, similar values were observed between both placebo and CsA groups with no significant differences and thus even in the putamen (Supplemental Fig. 5). Strikingly, the thalamus displayed a unique neuro-inflammatory pattern (Fig. 4). Δ DVR_D7−baseline_ in the posterior thalamus displayed a clear increase tendency regarding the placebo group compared to the CsA-treated group (posterior thalamus: 0.28 [−0.026, 0.35] vs. 0.048 [0.0029, 0.083] for placebo group vs. CsA-treated group respectively). At day 30, significant differences were measured in the placebo group compared to the CsA-treated group (anterolateral thalamus: 0.20 [0.064, 0.81] vs. −0.089 [−0.14, −0.045] for placebo group vs. CsA-treated group respectively, with *p* = 0.0022; posterior thalamus: 0.25 [0.084, 0.68] vs. −0.077 [−0.11, −0.049] for placebo group vs. CsA-treated group respectively, with *p* = 0.022).

**Fig. 5:**
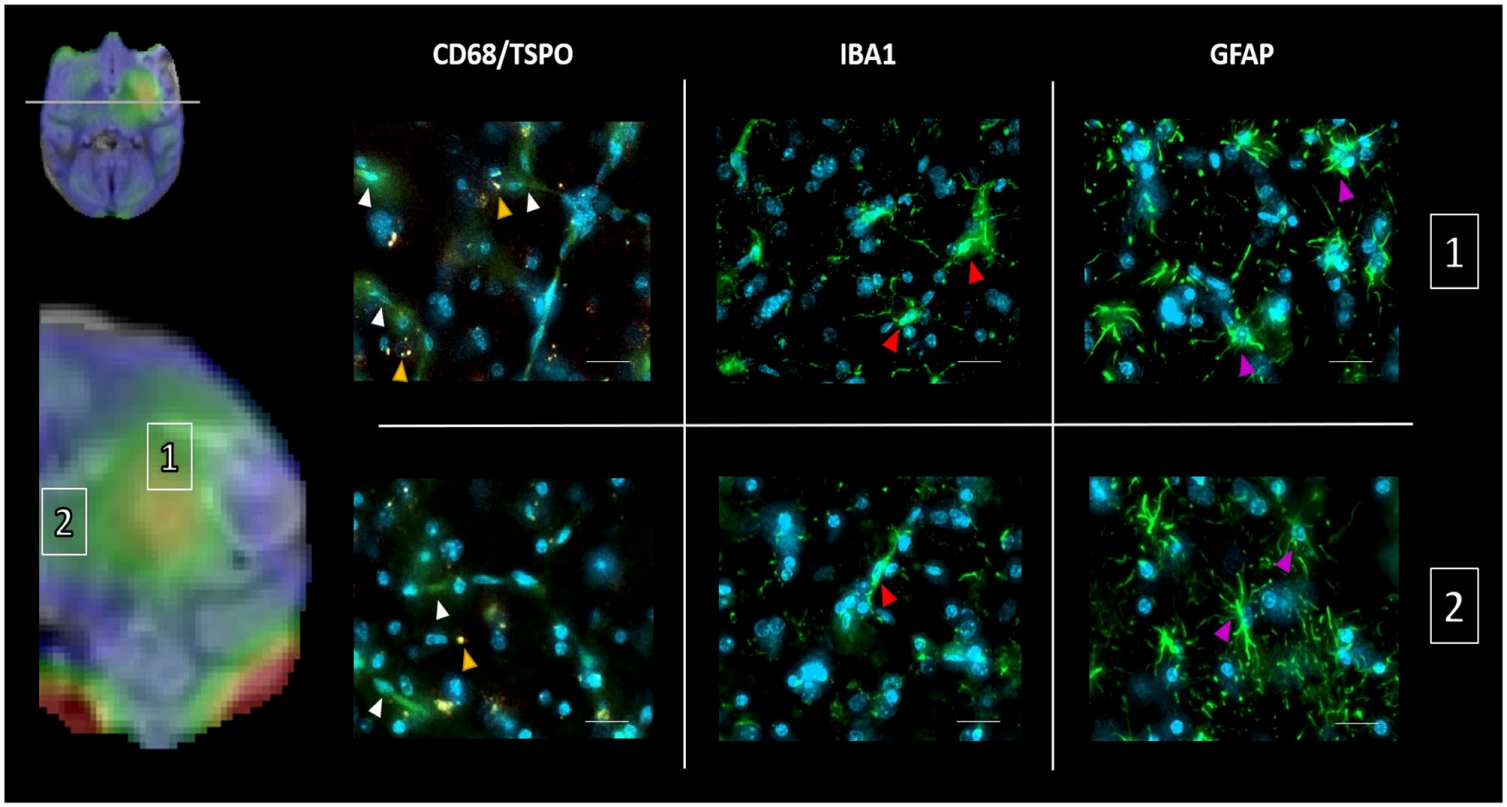
Post-mortem immunohistochemistry of corresponding in vivo PET-MRI (fluid attenuation inversion recovery MRI at day 30 and overlay of [^11^C]PK11195 DVR) (NHP #9). Tissue sections were double immunostained with CD68 for phagocytic cells (white arrow head) and TSPO for inflammation response (yellow arrow head). Tissue sections were immunostained with Iba1 for microglial response (red arrow head) or GFAP for reactive astrocytes (pink arrow head). All sections were counterstained with 4, 6-diamidino-2-phenylindole (DAPI, blue) for cell nuclei. **1**: peri-lesion area. **2**: thalamus. Scale bar: 50 m

### Immunofluorescence in Peri-Lesion Area and Thalamus

We confirmed the TSPO expression with immunolabeling and fluorescence in lesion and peri-lesion areas and in the thalamus (Fig. 5). The punctiform labeling, corresponding to mitochondria with high TSPO expression, was important in the peri-lesion areas as well as in thalamus regions. Simultaneously, we labeled the monocyte/macrophage CD68 marker and highlighted that the regions with high TSPO expression also displayed substantial CD68 expression. Microglia response and numerous reactive astrocytes were detected in peri-lesion and the thalamus, through Iba1 and GFAP markers respectively (Fig. 5).

## Discussion

Microglial reactivity following ischemic stroke injuries attracts constant interest as a therapeutic target. We reported here for the first time, to our knowledge, a longitudinal follow-up of microglial response in a minimally invasive stroke model mimicking EVT in NHP. First, using voxel-wise analyses with the baseline [^11^C]PK11195 database, we identified subjects with inflammation burden at day 30 (representing 36% in our study). Secondly, we described the dynamic of inflammation induced by stroke, characterized on the one hand by a focal brain inflammation of the cortical and subcortical lesioned regions (including the putamen), and by a global brain inflammation on the other hand. We confirmed the presence in our model of secondary thalamic injuries in relation to a specific pattern of inflammation in the thalamus. Finally, we brought indications in cyclosporine A-treated animals that early pharmacological protection of BBB permeability may allow to reduce remote inflammation.

### Longitudinal Imaging Approach of Inflammation on NHP After EVT Allowing to Study Inflammation Processes on the Whole Brain

Numerous neuroprotective drugs targeting inflammation have been considered in AIS as an adjunct to reperfusion therapies. But up to now, translation of neuroprotection from bench to bedside has failed [11]. To provide additional insights on post-stroke inflammation and to serve as an asset in future therapeutic evaluation of potential neuroprotective therapies, we develop a translational stroke model mimicking EVT in NHP. All animals had extensive longitudinal characterization of both ischemic lesions and inflammation. Based on the assumption that longitudinal measurement is not feasible in a clinical context, we build a database with all the [^11^C]PK11195 PET baseline scan to perform an individual voxel-wise analysis with day 7 and day 30 [^11^C]PK11195 PET scans, searching for an increased DVR at those timepoints compared to the baseline. Interestingly, no NHP displayed significant cluster of voxels at day 7 (Table 2). This may be due to the high inter-subject and intra-subject variability of [^11^C]PK11195 PET at baseline [24, 25]. Furthermore, our model displays a large interindividual variability in the volume of the damages, both during occlusion (lesion core measured with DWI and penumbra measured with PET) and at day 7 (established lesion assessed with FLAIR). As in humans, collaterals are important contributors as previously evaluated in this model [13]. At day 30, the identification of clusters of voxels with significantly higher inflammation compared to the baseline database in 4 animals confirmed the persistence of a focal inflammation in the lesion area. Most of the voxels with increased [^11^C]PK11195 DVR at day 30 are within the per-occlusion measured lesioned area (Fig. 2B and Supplemental Fig. 4), and few of the voxels are located in the penumbra. With the *Macaca fascicularis* atlas, we identified anatomical regions with persistent inflammation at day 30 and found the involvement of the posterior putamen for three of the four subjects. We have observed two general patterns of inflammation in our NHP model of CIR. The first one is the frequent matching of striatal inflammation and stroke lesion assessed by FLAIR MRI (Supplemental Fig. 2 NHP #3, #13). The second pattern is the presence of cortical inflammation. This pattern could even interest remote cortical areas like parietal cortex (Supplemental Fig. 2 NHP #5). Whether these patterns reflect two different processes related to lesional process in striatum and to compensatory mechanisms in cortical areas remains an open question. This will be investigated in further studies using extensive behavioral testing in relation to motor impairment for striatal lesion and cognitive processes for cortical compensatory mechanisms. Our data bring interesting information with regard to post-stroke rehabilitative therapy with potential specific test in relation to specific neuro-inflammation pattern. The frequent involvement of the striatum, and more precisely the putamen, is of interest given that it is irrigated by the middle cerebral artery, occluded in our study. Furthermore, subcortical structures like the striatum deserve in-depth studies given their dual implication, in clinical deficit manifestation as well as in functional recovery [26–28]. Therefore, the question whether the significant cluster in putamen may serve as an imaging biomarker either of lesion severity or of functional recovery will be tested in the abovementioned planned studies.

### The Cyclosporine Treatment Reduces Both the Focal and Global Inflammation

The present phenotypic characterization of inflammation process revealed that our model mimics clinical neuro-inflammation observed in patients [29]. This pattern is composed of a focal inflammation, defined as a localized region of inflammation overlapping the lesion core, and of a global neuro-inflammation. Although the dynamics of both phenomena vary in our model compared to clinical data, here we observed that global brain inflammation extension is maximum at day 7 and spatially regresses at day 30. In the recent review on clinical global brain inflammation, Shi and coll. reported that brain inflammation becomes globally distributed within months after the primary event [29]. This discrepancy may be due to interspecies variation and could be enhanced by the controlled experimental conditions which contrast with the higher clinical variability. The early invasion of peripheral myelomonocytic cells through a disrupted BBB is thought to participate to the inflammation process [30, 31]. The early protection of the BBB may reduce this infiltration and reduced brain damages, although the therapeutic window seems narrow as longer term effects of myeloid cells favorize angiogenic repair [31]. In the pericytes, the cyclophilin A pro-inflammatory pathway is linked to BBB breakdown, which phenomenon can be reversed by low dose of CsA [14]. The protection of neuro-vascular integrity thanks to CsA-induced inhibition of the cyclophilin A pathway in the pericytes has been shown to be neuroprotective [15]. This concept serves as rationale for our hypothesis of a protective effect of cyclosporine A (CsA) injected at the dose of 2 mg/kg 5 min before recanalization. In CIR injuries, CsA inhibits the mitochondrial permeability transition pore opening by binding with cyclophilin that locates in the inner mitochondrial membrane and may reduce cell death and DAMPS release [32]. We previously demonstrated that CsA injection allows to reduce the permeability of the BBB in our NHP model of stroke [16]. We observe in the present study a decrease tendency in inflammation measured by [^11^C]PK11195 PET in the CsA-treated group compared to the placebo group. This trend is observed at day 7 both in the lesion core and in the penumbra ROIs of the lesion, defined with DWI and PET-[^15^O]H_2_O perfusion imaging respectively [13]. At day 30, this decrease tendency was reduced for both ROIs, despite the significant difference observed between the placebo group and the CsA-treated group in the penumbra. This difference is obviously driven by one individual (# NHP7) with a distinct and specific inflammatory pattern (with global increase at day 30, Supplemental Fig. 5). Yet, microglia are the first to be on-site and initially stabilize damaged vessels [31]. However, their prolonged activation by DAMPS is a key contributor to spreading inflammation and secondary injury [33, 34]. Lower ADC at occlusion is the hallmark of irreversible damage and its overlap with the persisting inflammation cluster at day 30 in the putamen may reflect these microglia responses to DAMPS.

Brain repair and neuroprotective therapies would beneficiate from translational imaging biomarkers allowing patient stratification and clinical trial endpoint assessment [35]. From this translational perspective, our results revealing a spatial matching between the acute ADC drop and chronic inflammation in the putamen may be of clinical importance. As such, ADC value is a clinically available parameter and its below-threshold value in the putamen could be tested to stratified patients in neuroprotective trials targeting inflammation. Besides, iron-sensitive imaging with ferumoxytol has been shown as a relevant translational method to investigate inflammation using MRI, and could be an ideal complement for patient follow-up [36, 37].

### The Thalamus, a Key Subcortical Structure in Remote Inflammation After Stroke

Regarding remote inflammation, the thalamus is known as being particularly sensitive to secondary injuries [38, 39]. The mechanisms of this inflammation related to secondary thalamic injury is not yet fully understood [40]. It probably combines Wallerian’s degeneration of axons, connecting the lesioned cortical areas to the thalamus, that triggers microglial and astrocytic activation [41] as well as brain infiltration of peripheric immune cells and pro-inflammatory signaling cytokines during the acute phase [42, 43]. Importantly, the thalamus was never involved in the lesions, neither in acute diffusion-positive lesion/FLAIR-positive areas nor in the hypoperfused [^15^O]H2O PET-assessed penumbra, in none of included animals. Similarly, to what we observe in lesion and penumbra ROIs at day 7, the Δ DVR_D7−baseline_ measured in the posterior thalamus of the CsA-treated group is lower than the one measured in the placebo group, albeit this difference is not statistically significant. This difference persisted at day 30 and was even expanded to the anterolateral thalamus. As we discuss previously, the statistical inference must be interpreted with caution as it may be mainly driven by just one individual (#NHP 7, Supplemental Fig. 5). We qualitatively assessed reactive glial cells in representative individual using post-mortem immunohistochemistry (Fig. 5). We found similar pattern of Iba1 + microglia and reactive GFPA + astrocytes in peri-lesion and thalamic areas. Besides, we used CD68 immunofluorescence labeling to confirm the presence of a phagocytic activity in both areas 30 days after the transient MCAo [44]. The presence of phagocytic cells in secondary lesioned area during the chronic phase of our model suggests that the degeneration of cortico-thalamic axons is a phenomenon that lasts beyond the subacute phase in our NHP model of IR. As previously mentioned, MRI of iron oxide nanoparticles could be helpful to investigate in vivo the inflammation related to phagocytes’ activity [45]. Overall, our results support the therapeutic potential of BBB protective therapy during the acute phase of reperfusion to reduce brain inflammation, in both adjacent and remote regions to the lesion. We previously demonstrated that CsA injection at reperfusion reduced BBB leakage [16]. We suggest here that this reduced BBB leakage may lower stroke lesions which then require less microglial activation [46]. Further studies will have to investigate the profile of infiltrating immune cells and related cytokines in the areas adjacent to the lesion as well as in the remote areas. Moreover, studies are required to specify the role of microglia in secondary thalamic inflammation in our model, as it could reflect degenerative or remodeling processes [47–49].

Some limitations of the present study deserve mention. Firstly, the limited sample size might have reduced our ability to detect the effect of CsA on the focal inflammation in the lesion core and the penumbra. Especially at day 7, we described the decrease tendency in Δ DVR_D7−baseline_ between CsA and placebo-treated group (*n* = 6 and *n* = 5 respectively). Secondly, and in relation to the first point, the (*R*)-[^11^C]PK11195 radiotracer has a relatively poor signal-to-noise ratio. This led to the development of the so-called second-generation TSPO radioligands [50]. Although most of the ischemic stroke patients included in PET inflammation studies were scanned with (*R*)-[^11^C]PK11195 [51], it would be of interest to test a “second-generation” TSPO radioligand in our model to determine if their higher affinity and specific binding could balance the small sample size inherent in NHP model of transient MCAo.

Our NHP model of EVT offers the possibility to bridge the gap between rodent models and clinical context. This model presented a general inflammation at day 7 as a typical reactive process triggered by MCA occlusion. Moreover, we detected a high focal inflammation in 36% of the subjects at day 30, which highlight the individual response to acute ischemic stroke that might complement general response. Further studies using per-occlusion PET-MR imaging will characterize oxygen metabolism to build translational methodology that will complete the individual follow-up in a clinical context.

## Supplementary Information

Below is the link to the electronic supplementary material.

Supplementary file1 (PDF 1550 KB)

Supplementary file2 (PDF 985 KB)

Supplementary file3 (PDF 1568 KB)

Supplementary file4 (PDF 1549 KB)

Supplementary file5 (PDF 1545 KB)

Supplementary file6 (PDF 1546 KB)

Supplementary file7 (PDF 1553 KB)

Supplementary file8 (PDF 1546 KB)

Supplementary file9 (PDF 1561 KB)

Supplementary file10 (PDF 1554 KB)

Supplementary file11 (PDF 1561 KB)

Supplementary file12 (PDF 1540 KB)

Supplementary file13 (PDF 1557 KB)

Supplementary file14 (PDF 1542 KB)

Supplementary file15 (PDF 1543 KB)

Supplementary file16 (DOCX 45 MB)

## Data Availability

Datasets generated during this study are available from the corresponding author upon reasonable request.

## Abbreviations

ADC: Apparent diffusion coefficient
AIS: Acute ischemic stroke
BBB: Blood-brain barrier
CBF: Cerebral blood flow
CIR: Cerebral ischemia reperfusion
CsA: Cyclosporine A
DAMPs: Damage-associated molecular patterns
DCE: Dynamic contrast enhancement
DSC-MRI: Dynamic susceptibility contrast MRI
DVR: Distribution volume ratio
DWI: Diffusion-weighted imaging
EVT: Endovascular thrombectomy
FLAIR: Fluid attenuated inversion recovery
GFAP: Glial fibrillary acidic protein
HT: Hemorrhagic transformation
Iba1: Ionized calcium-binding adapter molecule 1
MCAO: Middle cerebral artery occlusion
NHP: Non-human primate
ROI: Region of interest
SPM12: Statistical parametric mapping, version 12
TSPO: Translocator binding protein
