# Supplementary material for: Spatio-Temporal Characterization of Brain Inflammation in a Non-human Primate Stroke Model Mimicking Endovascular Thrombectomy
Source: Neurotherapeutics. 2023 Mar 28;20(3):789–802. doi: 10.1007/s13311-023-01368-2 (PMC10275847; doi:10.1007/s13311-023-01368-2)
Supplement: Supplementary file 16 — Supplementary file16 (DOCX 45 MB) [file 13311_2023_1368_MOESM16_ESM.docx]

**Supplementary material**

**Spatio-temporal characterization of brain inflammation in a non-human primate model mimicking endovascular thrombectomy**

Guillaume Becker ^1*^ ; Justine Debatisse^1^ ; Margaux Rivière ^1^ ; Claire Crola Da Silva ^1^ ; Maude Beaudoin-Gobert ^2^ ; Omer Eker ^3,4^ ; Océane Wateau ^5^ ; Tae Hee Cho ^1,4^ ; Marlène Wiart ^1^ ; Léon Tremblay ^6^ ; Nicolas Costes ^7^ ; Inès Mérida ^7^ ; Jérôme Redouté ^7^ ; Christelle Léon ^1^ ; Jean-Baptiste Langlois ^7^ ; Didier Le Bars^†^ ^4,7^ ; Sophie Lancelot ^4,7^ ; Norbert Nighoghossian ^1,4^ ; Laura Mechtouff ^1,4^, Emmanuelle Canet-Soulas ^1^.

^1^ CarMeN Laboratory, INSERM U1060, INRAE U1397, University Claude Bernard Lyon 1, Lyon, France. ^2^ Lyon Neuroscience Research Center, CNRS UMR5295, INSERM U1028, Université Claude Bernard Lyon 1, Lyon, France. ^3^ CREATIS, CNRS UMR-5220, INSERM U1206, Université Lyon 1, INSA Lyon, Villeurbanne, France. ^4^ Hospices Civils de Lyon, Lyon, France. ^5^ Cynbiose SAS, Lyon, France. ^6^ Cognitive Neuroscience Center, CNRS UMR5229, Université Claude Bernard Lyon 1, Lyon, France. ^7^ CERMEP - Imagerie du Vivant, Lyon, France.

^*^ Corresponding author:

Dr. Guillaume Becker

Email: guillaume.becker@univ-lyon1.fr

Laboratoire CarMeN, INSERM U1060, Université Lyon1, INRAE U1397, Groupement Hospitalier Est, 59 Boulevard Pinel, 69500 Bron, France.

**Content:**

**Supplemental Table 1.** Lists of primary and secondary antibodies.

**Supplemental Figure 1.** Experimental design.

**Supplemental Figure 2.** ﻿[^11^C]PK11195 baseline database post-processing.

**Supplemental Figure 3.** Additional individual dataset.

**Supplemental Figure 4.** Cluster’s maps overlays.

**Supplemental Figure 5.** Quantification in Putamen.

**Supplemental Figure 6.** Specific evolution of inflammation in NHP #7.

**Supplemental Table 1.** Lists of primary and secondary antibodies.

| Primary antibody | Catalog # | Company | Host species | Clonality | IHC dilution |
| --- | --- | --- | --- | --- | --- |
| TSPO | LS-B14234-50 | Cliniscience | rabbit | polyclonal | 1:1000 |
| CD68 | ab201340 | abcam | mousse | monoclonal | 1:100 |
| GFAP | Z0334 | DAKO | rabbit | polyclonal | 1:500 |
| IBA1 | 019-19741 | WAKO | rabbit | polyclonal | 1:2000 |
|  |  |  |  |  |  |

| Secondary antibody | Catalog # | Company | IHC dilution |
| --- | --- | --- | --- |
| AlexaFluor goat anti-rabbit 568 |  | Invitrogen | 1:1000 |
| AlexaFluor goat anti-mouse 488 |  | Invitrogen | 1:1000 |
| AlexaFluor goat anti-rabbit 488 |  | Invitrogen | 1:2000 |
| AlexaFluor goat anti-rabbit 488 |  | Invitrogen | 1:2000 |

**Supplemental Figure 1.** Experimental design. The endovascular MCA occlusion (MCAo) was initiated in the angiography suite. Afterward, the animals were transferred in the PET-MRI scanner for data acquisition. The occlusion time was 110 minutes. CsA or placebo were injected 5 minutes before recanalization (black arrow head).

Spatial normalisation / smoothing

**Supplemental Figure 2.** ﻿[^11^C]PK11195 baseline database post-processing pipeline and days 7 and 30 voxel-wise analysis.

**Supplemental Figure 3.** Additional individual dataset.

**Supplemental Figure 4.** Mapping of inflammation clusters at day 30 (blue), stroke core lesion (red) and ischemic penumbra (green) during occlusion (overlaid on FALIR D30).

**Supplemental Figure 5.** Quantification in Putamen. Individual values of absolute differences in [11C]PK11195 DVR. Differences between the baseline and day 7 (*Δ* DVR_D7 – Baseline_). Differences between the baseline and day 30 (*Δ* DVR_D30 – Baseline_). Placebo n = 5, CsA n = 6.

**Supplemental Figure 6.** Specific evolution of inflammation in NHP #7.
